# How Experiences of Stigma and Discrimination at Various Points in the Queer Life Course Interrelate with Alcohol Use: Views from Sexually and Gender Diverse Youth

**DOI:** 10.3390/ijerph21111472

**Published:** 2024-11-06

**Authors:** Jorge Flores-Aranda, Yannick Gaudette, Anthea Dalle, Dominic Nadeau, Mathieu Goyette

**Affiliations:** 1 School of Social Work, Université du Québec à Montréal, Montreal, QC H2L 4Y2, Canada; gaudette.yannick@uqam.ca (Y.G.); nadeau.dominic@uqam.ca (D.N.); 2 Sexology Department, Université du Québec à Montréal, Montreal, QC H2L 4Y2, Canada; dalle.anthea@uqam.ca (A.D.); goyette.mathieu@uqam.ca (M.G.)

**Keywords:** LGBTQ+ youth, alcohol use, queer life course, stigma, discrimination

## Abstract

Background: The life courses of sexually and gender diverse individuals are shaped by a series of events that include acceptance of one’s own sexual orientation or gender identity, the coming out process and socialization in the LGBTQ+ (for Lesbian, Gay, Bisexual, Transgender, Queer/Questioning, and other sexual orientations and gender identities represented by the plus sign) environment. Generally experienced in a cis-heteronormative context, this process is marked by stigma and discrimination and the social harms they can cause, including a higher-than-average prevalence of alcohol use. Objectives: To profile the alcohol use of sexually and gender diverse (SGD) youth from a life course perspective and to explore individual perceptions regarding the personal, social and cultural factors (including stigmatization and its consequences) modulating their consumption. Methodology: This qualitative descriptive study is grounded in symbolic interactionism. Semi-structured interviews lasting approximately 90 min were conducted with LGBTQ+ youth aged 18 to 30 using alcohol at least once a week. A thematic analysis was performed. Results: A total of 31 individuals aged 18–29 (average age: 25) were interviewed. The average score regarding alcohol use was 14.25 (Standard Deviation -SD-: 4–31), which corresponds to a moderate risk and indicates the need for a brief intervention. Our study documents how the higher alcohol use among LGBTQ+ youth is shaped by individual, community and cultural factors at different points in the queer life course. Among the factors influencing drinking are the emotions experienced when questioning sexual orientation and/or gender identity as well as the feelings resulting from stigma and discrimination. Our findings also indicate the influence of socializing in the queer community and meeting peers and partners, as well as that of LGBTQ+ cultural practices. Discussion: Our study indicates the need for grassroots-level interventions that work to mitigate social pressures in queer socialization contexts. Accordingly, any intervention, whether preventive or therapeutic, must consider the interplay of personal, social, community and cultural factors. Interventions regarding alcohol use must build on the strengths of community and the sense of belonging.

## 1. Introduction

### Alcohol Use and the Life Courses of Sexually and Gender Diverse (SGD) Youth

The life courses of sexually and gender diverse (SGD) individuals are coloured by a series of events that include acceptance of one’s own sexual orientation or gender identity, the coming out process and socialization in the LGBTQ+ environment and having to confront social norms concerning sexual and gender diversity, personal perceptions of these social norms, and sexual behaviours [1,2]. All of these are key milestones in what can be termed the queer life course. Generally experienced in a cis-heteronormative context, it is marked by stigma and discrimination and the social harms they can cause, including a higher-than-average prevalence of substance use. Despite advances in civil rights in Canada for people of all genders and sexual orientations, discrimination and stigmatization persist [3].

Sexually and gender diverse (SGD) individuals of all ages have reported higher rates of alcohol use compared to the general population [4,5]. Various studies indicate significant alcohol use disparities between SGD youth (ages 18–29) and their heterosexual and cisgender peers [6,7,8]. A meta-analysis of the relationship between sexual orientation and adolescent substance use has shown the use of psychoactive substances, including alcohol, to be almost three times higher among young people identifying as gay, lesbian or bisexual than among their heterosexual peers [9]. Some studies suggest that SGD youth are proportionally more likely than their heterosexual and cisgender counterparts to engage in episodes of risky drinking, including binge drinking, defined as consuming a large number of alcoholic beverages in a short space of time [10] or surpassing the recommended limits [9].

Among SGD youth, the use of psychoactive substances, including alcohol, is influenced by a complex array of personal, cultural, social and structural factors specific to this population [6]. Studies have established links between the psychoactive substance use of SGD individuals and the minority stress they experience [11,12], arguing that people from sexual minorities are subject to higher rates of stress than the rest of the population, in large part due to sexual stigma [13,14]. In this context, the risk factors for substance use are often linked to victimization and discrimination as well as to such unique stressors such as internalized homophobia or transphobia [10,15], all of which are significant sources of social harm. Research documenting the risks of hostile social settings also correlates school-based discrimination and victimization with a heavier use of psychoactive substances, including alcohol, by lesbian, gay, bisexual and transgender adolescents [16].

However, the current literature on alcohol use among LGBTQ+ people yields few findings as to the links between the queer life course and alcohol use in particular [17]. The focus on sexualized substance use in this population has also resulted in less attention being paid to the specific role of other substances like alcohol [18]. In this sense, it is important to note that this project was funded as part of a competition specifically focused on alcohol consumption

To fully understand the possible interrelationships between the life courses of SGD individuals and alcohol use, we need to understand both the significance of drinking in queer communities and the way in which aspects of the queer life course, including stigmatization and any resultant social harms, shape individual consumption. Our aims are therefore (1) to profile the alcohol use of SGD youth from a life course perspective and (2) explore individual perceptions regarding the personal, social and cultural factors (including stigmatization and its consequences) modulating their consumption.

## 2. Materials and Methods

### 2.1. Research Design and Theoretical Approach

Our study uses a qualitative descriptive approach [19,20] to examine the meanings ascribed to alcohol use by SGD youth throughout their queer life course. The study is grounded in symbolic interactionism, a theoretical framework focused on the interrelationships between individuals and their society as well as the factors that modulate these interactions. Under this approach, meanings are understood through inductive research that involves direct contact with the study subjects [21,22]. Both the experience of alcohol use and the life courses of SGD youth are part of a social context modulated by the norms and values that arise from everyday interactions between people, ideas and events. In this sense, symbolic interactionism appeared to us as an appropriate framework for our chosen topic.

### 2.2. Sample and Recruitment

Our sample consisted of 31 youth aged 18 to 30 who consumed alcohol at least once a week. This criterion was based on the need to document the meanings assigned to “regular” alcohol use, including consumption considered non-problematic (low risk) [23], as well as those ascribed to people who chose to drink more often. Indeed, according to the World Health Organization (WHO), under certain conditions, drinking once a week or even more can still represent low-risk alcohol consumption [23]. Furthermore, we used broad selection criteria because we wanted to include individuals with a wide range of experiences regarding alcohol use and the queer life course. The sample age range was determined based on the fact that, in LGBTQ+ communities, substance use tends to continue into the twenties or even beyond as well as taper off less pronouncedly with age, compared to heterosexual groups [24].

Two SGD individuals with lived experiences of alcohol use advised the team as to the recruitment strategies to favour. To reach the study population, a recruitment poster was published on the TRADIS Canada Research Chair’s social media pages (Facebook, Instagram). Community organizations providing services to SGD youth relayed information about the study to the community. Furthermore, inspired by snowball sampling methods [25], study participants were invited to share information about the project through their networks.

### 2.3. Data Collection and Analysis

Data were collected between May and November 2023 through semi-structured interviews lasting approximately 90 min. These were carried out either in person or via a videoconference (Zoom) and were recorded and transcribed in full. Recordings were destroyed after transcription. The data were then anonymized and analyzed. The data are stored on the university’s cloud solutions and will be deleted five years after the end of the study. Interviews were conducted in either English or French, and transcriptions were made in the language in which the interviews were conducted. For the verbatim extracts from the French interviews presented in this article, translations have been provided.

The interview guide consisted of open-ended questions regarding alcohol consumption trajectories, the life courses of people whose sexual orientation and/or gender identity did not align with heterosexual or traditional gender norms (queer life course) and the perceived interrelations between these life courses and alcohol use. Here are some examples of questions: I would like you to tell me how your alcohol consumption began and how it has evolved over time; I would like you to talk about your life course as a young person from the sexual and gender diversity community; I would like you to describe the relationship between your alcohol consumption and your life course as a young person from the sexual and gender diversity community; Throughout your life course, how has your sexual orientation or gender identity modulated (or not) your alcohol consumption?

Following the interviews, participants were asked to complete a sociodemographic questionnaire along with the Alcohol, Smoking And Substance Involvement Screening Test (ASSIST) [23] so that we could create substance use profiles. The questionnaire addressed the topics of age, sexual orientation, gender identity, relationships, education, income and ethnic/cultural background.

Consistent with the study’s theoretical basis, our interview content was subject to ongoing thematic analysis, i.e., as data collection progressed [26]. The analysis made it possible to identify different topics, grouped under headings based on the interview guide, as well as emerging themes. Thematic clusters were subsequently identified based on characteristics such as recurrence, divergence, convergence and complementarity [26]. Approximately 10% of the material was co-coded by two of the study’s authors under the supervision of a third [26,27]. The analysis was performed using NVivo 12 software (QSR International, Melbourne, Australia). Data from the ASSIST tool were input into IBM SPSS Statistics 27 software (IBM, Armonk, NY, USA) and subject to an average’s calculation, based on the questionnaire’s score analysis guidelines [23]. The ASSIST results pertaining to the risk levels associated with alcohol use were categorized into three groups relative to the score obtained. A score of 0–10 indicated low-risk consumption; 11–26, risky drinking and the need for brief intervention; and 27 or higher, the need for specialist assessment and treatment. Through this calculation, we were able to classify participants’ levels of alcohol and other substance use and gauge any potential need for intervention.

### 2.4. Ethical Considerations

This project has been approved by the Institutional Committee on Ethics in Research Involving Humans at the Université du Québec à Montréal. The approval number is 22-4681. After the interview, the interviewers took a moment to check in with the participants about how they felt. Additionally, we provided each participant with a list of resources for support if needed. Finally, the interviewers were trained in social work, which gave them the ability to intervene if necessary

## 3. Results

### 3.1. Sociodemographic Characteristics

A total of 31 individuals aged 18–29 (average age: 25) were interviewed. Our questionnaire posed open questions about sexual orientation and gender identity, leaving participants free to identify with more than one (Table 1 and Table 2). Regarding place of residence, the majority were based in a large urban centre; several participants had relocated there from rural areas. Most identified as being of Caucasian origin; those who were born outside of Canada were from Europe, the Middle East and Latin America. Regarding education, the majority had an undergraduate university degree.

### 3.2. Participants’ Alcohol Use Profiles

The average score in our sample regarding alcohol use was 14.25 (SD: 4–31), which corresponds to a moderate risk and indicates the need for a brief intervention. Table 3 presents the sample in terms of percentages representing the level of intervention they should receive. According to our findings, more than half would require a brief intervention in relation to their scores, with a further 9.6% showing the need for intensive treatment. Participants received essential information regarding the services they could access in relation to their alcohol use.

### 3.3. Perceptions of SGD Youth Concerning Individual, Social and Cultural Factors Modulating Alcohol Use Throughout the Queer Life Course

Our sample associated their motivation to drink with individual, social and cultural factors that they linked to different points in their queer life course. The individual factors raised included using alcohol to navigate difficult emotions as well as to explore their sexual orientation or gender identity. Many of the emotions described were directly linked to their experiences of stigma and discrimination.

Regarding social factors, some participants linked their alcohol use to their ties with the queer community, including socializing with peers and meeting sexual or romantic partners. It is important to note here that feeling oneself to be part of the community could also promote a feeling of security. Accordingly, ties with the queer community could modulate consumption upwards or downwards.

Lastly, in terms of cultural factors, a number of participants associated alcohol use in the queer community with the prevalence of alcohol both at community cultural events and in cultural products aimed at LGBTQ+ populations.

#### 3.3.1. Alcohol as a Coping Strategy for Difficult Emotions Associated with Certain Points in the Queer Life Course

Several participants reported that their use of alcohol was motivated by the need to cope with different emotions, particularly those associated with the process of determining their sexual orientation and/or gender identity. This was the case with Léon, whose alcohol use had increased during periods when he was questioning his sexual orientation. Later in the interview, he specified that his drinking had decreased after these periods.

*I think I had a lot of questions [about my sexual orientation], particularly when I was a teen. So, yes, these times of questioning possibly led me to drink more. Precisely because [alcohol] helps loosen inhibitions and so I felt freer to be who I wanted to be. It was more between the ages of 18 and 24, kind of a time of questioning, of encounters*.—Léon, 26, gay, gender nonconforming.

Some participants felt that stigma and discrimination stemming from the heteronormative social context could give rise to uncomfortable emotions that in turn affected their alcohol use. This was the case with Ambre, who linked her alcohol use to anxiety and the invisibility she had experienced in relation to her sexual orientation.

*Bottom line, if you take my drinking, which is linked to my anxiety, well, part of my anxiety can be explained by the things I’ve had to suffer in life, like being invisibilized.*—Ambre, 26, lesbian/queer, cisgender woman.

Alcohol was also used to cope with the feelings engendered through experiences of gender identity-based discrimination. For Félix, who described the discrimination he suffered at the start of his hormone therapy, what was particularly distressing was being misgendered, though he also noted that these incidents began to diminish as the results of his treatments became more apparent.

[May 2022 was] *when I started hormone therapy. In the months leading up to it, I drank quite a bit, but once the [hormone] therapy started, I decided to cut back. A few months later, maybe three months…with hormone therapy, it can take three to six months before you see changes in your body, and so at that point I still couldn’t see any. And so being misgendered and so on…I found it really hard, so I drank more.*—Félix, 22, pansexual, trans man

Similarly, alcohol could be used to palliate the negative emotions associated with the distress of gender dysphoria. This was the case with Pauline, who reported drinking more to mitigate the dissociation she felt at a certain point in her life from feeling at odds with her assigned gender.

*I didn’t realize it at the time, but in hindsight, yes, [gender identity] had an impact. Basically, the gender dysphoria I felt, which was experienced mainly as dissociation, was less intense when I was drunk, and I found I liked being drunk precisely because it numbed me*.—Pauline, 26, bisexual, trans woman.

Alcohol use could also serve as a strategy for managing the fears associated with the coming out process. Victor describes how their drinking increased at this time, in large part due to what they anticipated as the consequences of disclosing their sexual identity.

*My drinking spiked when I was around 15/16, a time when it seems to me I was dealing with coming out. I had all of this to deal with and so I think alcohol was just a facilitator in that sense, helping me cope with all the negative feelings that can be associated with that.*—Victor, 27, queer, non-binary.

#### 3.3.2. Alcohol Use and Exploring Sexual Orientation and Gender Identity

The exploration of gender identity modulated the alcohol use of some participants. As Louna reported, their alcohol consumption would fluctuate depending on how they performed their gender. Indeed, they noted that they drank more heavily when feeling the need to perform a gender closer to what was considered “masculine,” but did not drink at all when performing their gender in a way seen as less so.

*My use of alcohol was closely tied to wherever I’d situated myself on the spectrum of masculine and feminine. When I tried to put myself on the masculine end, I drank a lot, and it was because it was linked to an indirect performance of “maleness.” I never articulated it to myself like that, but that’s what it was… When I began to get in touch with my feminine side by coming out as gay, showing my twinkness, well, I didn’t drink. It’s to do with perception… Or at any rate, the way in which I had internalized our society’s gendered tropes. The masculine side is very impulsive, very out of control, whereas the feminine is all about restriction and control. This applies just as much to my journey with food as it does to drugs or alcohol.*—Louna, 25, homosexual, non-binary transfeminine.

Alcohol could also facilitate the exploration of sexual orientation. For some, drinking played an important role inasmuch as it made it easier for them to develop intimate relationships with a person of the same gender.

*I went out more with guys only because I’m not very good at seducing women. So* [with] *women, there were more new experiences. I drank more* [when socializing] *with women just because it was new to me… It was associated with newness, and so it turned out that what was new for me was having* [intimate] *relations with women.*—Mylène, 27, bisexual-pansexual, cisgender woman.

#### 3.3.3. Alcohol Use and Community Connectedness

Some participants cited their relationship to the LGBTQ+ community as influencing their alcohol use. While a number linked increased drinking to becoming part of the community, the situation appeared more nuanced for others, who associated it with peer pressure, i.e., when in group situations, particularly in festive settings:

*I’d say the main thing is, when I go out with friends, that’s the only time I feel pressured to drink.*—Dan, 29, homosexual, cisgender man.

On the other hand, a lack of access to the LGBTQ+ community could in itself exacerbate alcohol use. One participant reported that her drinking intensified when, during her studies, she had lived in a rural area. Struggling to form a queer circle of friends, she found herself socially isolated and thus more prone to drink.

*Because there was no community. I had no friends in the community, so I was more isolated. I had more opportunities to drink.*—Gabriel.le, 18, lesbian, non-binary.

A similar situation emerged for another participant named Happy, for whom the feelings of aloneness engendered by immigrating to Canada intensified their alcohol use. They also had to learn the social codes of their new country’s queer community before being able to assimilate.

*If drinking was already problematic for me, it was in part linked to general life problems and maybe a feeling of being alone. Then I came to Canada and had no community to land in and didn’t know how to find one, so that fed into the feeling of being alone. And for sure it can affect* [alcohol use]*. For example, not knowing how to approach a woman because… In a country where* [homosexuality] *is illegal, the lesbian/bi scene is pretty limited, and I can tell you, there is no approaching. It’s* [laughs]*… in my country you just feel it [laughs], you recognize each other, you go for it. Whereas* [in Canada]*, it’s a whole new game, so that definitely had an impact in the sense that there was a learning curve.*—Happy, 26, pansexual, non-binary.

While increased alcohol use could be linked to contact with the queer community, including while socializing in festive LGBTQ+ settings, participants also reported that those same settings could generate feelings of security, given that they represented safe spaces to be in a festive context, as Stéphanie explained.

*I feel safer* [in queer bars]*. It’s been so long since I’ve been to a bar that made me feel uneasy because after so many years here, I know where my spots are… I remember being in places where I wouldn’t want to turn my back on anyone for too long, where I knew of many people having* [drugs] *dropped into their drinks.*—Stéphanie, 24, lesbian-queer, cisgender woman.

#### 3.3.4. Meeting Partners as a Modulator of Alcohol Intake

Some participants found that drinking made it easier to meet intimate partners by lowering certain inhibitions. For example, for Roxane, the “freeing” effect of alcohol allowed her to approach women. She also felt that approaching others with the intent to flirt or hook up was easier for men.

*It’s easier for me to hit on people after a few drinks because alcohol loosens me up. Like, I’ll be more at ease approaching a girl. For men, it just seems easier, even sober, because everyone sees it as “normal.”*—Roxane, 26, bisexual, cisgender woman.

Meeting peers and intimate partners who share the same gender identity was also seen to modulate participants’ use of alcohol. In this regard, a trans person reported that when she met another trans person, her alcohol consumption had decreased because she no longer felt the strain of having to explain her reality to a cisgender person.

*This is my first relationship with a trans person. It makes me feel good. You don’t have to explain the things you’d have to explain to a cis person. I feel totally comfortable expressing my masculine side, my feminine side, whatever. I drink less because I don’t have to get out of my own body.*—Vandy, 24, queer, trans man.

#### 3.3.5. The Role of Alcohol in LGBTQ+ Culture

Participants saw socialization and culture in LGBTQ+ environments as among the main factors influencing their alcohol use. Festive spaces associated with LGBTQ+ culture, particularly bars, become meeting places that not only made it possible to become part of the commu3nity, but also to assert one’s identity within it, as one participant who had immigrated to Montreal from a small European municipality explains.

*You try to do as much as possible, see as many people as possible. I think that the fact of being in the community means I go out more. In part, because I want to show that I’m here in the Village. But also because it’s* our *space, and we need to have this space… That’s also why I came here, because the Village is known all around the world. So, for sure I want to go out in the Village.*—Raphaël, 28, homosexual, cisgender man.

Some participants commented on the prevalence of alcohol in certain cultural products aimed at LGBTQ+ communities, such as TV shows.

*I’m rewatching certain seasons of* RuPaul’s Drag Race*. Alcohol use is very much in evidence. So that plays into things. Did it influence me? I’m not sure it did so directly. But it’s obvious to anyone watching that* [drinking is] *normalized in a gay or LGBT social context.*—Léo, 29, homosexual, agender.

Participants also commented on bars featuring drag shows that proposed “fun” activities inciting the audience to drink.

*Drag queens often drink a lot and can also incite others to drink. You know, like drinking games involving rounds of shooters or giving away free shooters or just reminding people all the time to drink alcohol*.—Marc, 28, homosexual, non-binary.

## 4. Discussion

Our study contributes to the current knowledge by documenting how the higher alcohol use among SGD youth is shaped by individual, community and cultural factors at different points in the queer life course. Among the factors influencing drinking are the emotions experienced when questioning sexual orientation and/or gender identity as well as the feelings resulting from stigma and discrimination. Our findings also indicate the influence of socializing in the queer community and meeting peers and partners, as well as that of LGBTQ+ cultural practices.

### 4.1. Stigma and Discrimination at Different Points in the Queer Life Course and Their Influence on Alcohol Use

Several studies have established a link between questioning (the process of exploring sexual orientation and/or gender identity) and increased alcohol consumption among SGD youth [4,9,28]. Our study nuances this by documenting that, once these periods of questioning have ended, alcohol use dwindles, at least for some people. Other studies describe alcohol use in this population as an outcome of being an outlier in a cis-heteronormative society—a situation giving rise to negative emotions that some choose to subsume with substances [13,29]. Our study corroborates these findings. However, our participants also reported how finding their community has helped them cope with cis-heteronormativity and, in some cases, lower their alcohol consumption. These observations are consistent with the Minority Stress Model, which suggests that queer communities also possess strengths in the face of stigma and discrimination, such as social support, community consciousness, identity pride, self-esteem, resilience, mental health, and positive health behaviors [30]. Our data allow us to observe that the community connections established by the participants facilitated an awareness of the support they could receive from their community.

We can discuss our results in terms of queer life courses. Although the participants are young, they reported information that illustrates the evolution of their queer life trajectory, including questioning their sexual orientation or gender identity, coming out, and meeting peers in their community, among other experiences. This is consistent with the framework of sexual orientation development over the life course [2]. It is important to note that the major milestones in this framework correspond to queer life trajectories in the West. However, some participants had a migratory background, and it is possible that these milestones differed for them.

### 4.2. Socialization, Queer Culture, Meeting Peers and Partners and Alcohol Use

Many studies document the prevalence of alcohol in queer social settings, a context they say encourages alcohol use among SGD people [17,31,32]. Furthermore, some studies maintain that, contrary to what has been observed in the heterosexual population, the queer life course fosters a prolonged use of alcohol and other psychoactive substances along with a tendency to stop only later in life, in large part due to the continued use of social settings where alcohol is present [33]. Our study adds a degree of nuance to these findings. On the one hand, participants confirmed the prevalence of alcohol in these environments, affirming that drinking can promote a sense of “fitting in” and make it easier to meet people, including sexual or romantic partners. On the other, they also saw alcohol use as part of the imposed norms specific to the queer environment. In this sense, the generally higher use of alcohol observed among our study sample could be experienced at different points in the queer life course as either supporting personal needs and desires or running counter to them. The stigma that participants might undergo would no longer be necessarily linked to their sexual orientation or gender identity, but rather to whether or not they conformed to community drinking norms. Still, it must be noted that these experiences were not uniform across our sample. For people from immigrant backgrounds or who came from rural regions, the process of integrating into the Montreal queer community could be more difficult since many lacked a social network there to begin with and may not know the social codes within queer communities.

Moreover, the participants discuss the support they receive from their community but do not address whether they have support from their family. This observation is consistent with the literature regarding queer youth, which indicates that, in the West, queer youth have more support from their families compared to their peers from previous generations [30].

### 4.3. Toward Interventions Promoting Low-Risk Consumption and Prevention

Like other studies, our research highlights how social discrimination against people from sexual and gender minorities affects their alcohol use [34,35] and points towards the need to promote a welcoming environment at different times of their lives and reduce minority stress [3]. Furthermore, as noted previously, drinking norms in the queer community can give rise to the intra-group stigmatizing of those who do not conform. Given the importance of queer social settings for fostering integration and feelings of belonging, it is important to consult with the community as to the most appropriate ways of promoting lower-risk forms of consumption. While some parts of the queer community see the role of substance use as a part of a subculture that enables the transmission of LGBTQ+ norms and values [36,37], others see it differently and call for the development of sober spaces for socializing [38,39]. Clearly, we are due for a community-wide conversation on the social norms that influence substance use as well as the stigma and social harms these could induce.

### 4.4. Limitations

This study has certain limitations, particularly the fact that the questions focused on past experiences, which may be influenced by memory bias. It is also a study that only examined alcohol consumption, while many individuals concurrently use other substances. It is important to point out that a small proportion of our sample had an immigration experience (particularly from Europe), which may have influenced their queer life course. Future studies by our team will examine the specific experiences of these individuals more closely. Additionally, it is a cross-sectional project, which did not allow us to see whether participants experienced changes in their queer life trajectories.

## 5. Conclusions

Together, these aspects indicate the need for grassroots-level interventions that work to mitigate social pressures in queer socialization contexts. Changing these norms could allow SGD individuals to make personal choices regarding their substance use without having to forfeit a sense of community belonging. Furthermore, alcohol use among SGD individuals is often overlooked by public and community organizations in favour of other substances and practices, particularly sexualized substance use. Accordingly, any intervention, whether preventive or therapeutic, must take into account the interplay of personal, social, community and cultural factors. Lastly, interventions regarding alcohol use must build on the strengths of community and the sense of belonging.

## Figures and Tables

**Table 1:** Sexual orientation (non-mutually exclusive answers).

Sexual Orientation	Frequency	%
Homosexual/Gay	7	22.6
Lesbian	5	16.1
Pansexual	9	29
Bisexual	9	29
Queer	6	19.4

**Table 2:** Gender identity (non-mutually exclusive answers).

Gender Identity	Frequency	%
Cisgender woman	13	41.9
Cisgender man	5	16.1
Non-binary	8	25.8
Transmasculine	2	6.5
Transfeminine	1	3.2
Trans man	3	9.7
Trans woman	1	3.2
Agender	2	6.5
Gender nonconforming	1	3.2

**Table 3:** Participant percentages per level of intervention indicated by the ASSIST.

	No Treatment	Brief Intervention	Intensive Treatment
Alcohol use in the last three months	32.2%	58%	9.6%

## Data Availability

The original contributions presented in the study are included in the article, further inquiries can be directed to the corresponding author/s.
